# Effectiveness of Mellow Parenting on parental mental health and parenting outcomes on a vulnerable parent sample in Moldova

**DOI:** 10.1371/journal.pone.0325245

**Published:** 2025-06-02

**Authors:** Ailsa Jones, Lisa Golds, Natalie Duncan, Irina Spivacenco, Daniela Mamaliga, Rachel Tainsh, Raquib Ibrahim, Angus MacBeth

**Affiliations:** 1 School of Health in Social Science, University of Edinburgh, Edinburgh, United Kingdom; 2 Mellow Parenting, Glasgow, United Kingdom; 3 Partnership for Every Child, Chișinău, Moldova; Universiti Putra Malaysia, MALAYSIA

## Abstract

**Introduction:**

Poor parental mental health is a risk factor for reduced mental health outcomes and increased behavioural problems in children. High quality parenting interventions are important in minimising the risks associated with poor outcomes for children.

**Aims and research question:**

This study assessed the implementation of Mellow Parenting, which provides a range of attachment-based parenting programmes, designed to help parents improve their capacity for sensitive and responsive caregiving and develop their relationship with their child, in Moldova. Research questions were 1) do parents who attend Mellow Parenting experience improvements in their wellbeing? 2) do parents demonstrate improvements in parenting confidence? and 3) do parents experience improvements in parenting daily stress?.

**Methods:**

Secondary data analysis was used for measures collected pre- and post-intervention from groups run in Moldova between 2016 and 2020 of n = 244 mothers. Outcomes were parenting wellbeing, parenting daily stress, parenting confidence, and children’s behaviour. The study focused on two of the current MP programmes; Mellow Babies and Mellow Toddlers.

**Results:**

T-tests were performed to assess effectiveness of treatment. Correlations and ANCOVAs explored the interactions between variables. Mothers who participated in the group showed improvements in their self-reported wellbeing, parenting confidence, and child behaviour. A reduction was seen in parental stress. Urban and rural intervention groups showed significant differences in terms of pre- and post-scores for anxiety, outward irritability, and parenting confidence.

**Conclusion:**

Mellow Parenting appears to be an effective intervention for mothers in Moldova in terms of improving parenting wellbeing and parenting confidence and reducing stress. The results of this study suggest that Mellow Parenting is an effective parenting intervention with potential for scaling up across Moldova, as well as other culturally similar countries across the Eastern European and West Asian regions.

## Introduction

How a child is cared for is fundamental to optimal developmental outcomes. Consistent parenting, a positive parenting environment, and a child-centred empathic approach are critical factors in mediating that child’s development and future success [1,2]. There is substantial evidence that the quality of interaction that a child has with their caregiver has a positive effect on infant mental health, with life-long lasting effects on the child in terms of social, cognitive and emotional development and mental health adjustment. The quality of the parent-child relationship has been associated with greater child independence, resilience, and improved social adjustment [3,4]. Secure attachment also allows children to build healthy relationships and to learn emotional regulation [5,6]. These benefits persist over time with longitudinal studies demonstrating that responsive parent-child relationships observed in secure parent-child attachments are associated with higher educational attainment, higher socio-emotional development and fewer behavioural problems [7,8]. One such study [9], reported that adolescents experiencing high quality interactions with their parents were statistically more likely to report higher ratings of parental warmth, effective behavioural management, and sensitivity. Maternal sensitivity also appears to have a long-term impact in protecting the child from internalising problems [10]. Factors related to parental wellbeing; mental health problems, drug dependency and domestic violence, can have an adverse effect on the relationship between parent and child [11–13]. This can hinder not only the attachment relationship, but also a parent’s ability to be attuned to their child’s needs [2]. Investment in early childhood development has been accepted worldwide as vital in ‘human capital development’ [14], but investment in the child is very closely allied to investment in their parents.

Compromised parental mental health can affect the quality of parenting and in turn, the development of the infant or child [15]. The consequences on the child are far-reaching; poor maternal mental health outcomes have been associated with poorer physical and mental health in children and young people, internalising and externalising behaviour problems, and poorer academic performance [16,17]. Maternal depression can impede typically responsive interactions, such as vocal exchanges between mother and baby, often described as the ‘serve and return’ of parent-child interaction and can impair the development of an infant’s brain architecture and stress response system [18]. Similarly, parental self-efficacy is important in moderating both parent and child outcomes. Self-efficacy denotes the caregiver’s confidence in how their parenting can influence their child in a ‘health and success-promoting manner’ [19]. Poor parental mental health can also affect the parent’s perception of themselves as an effective caregiver, further influencing the relationship with their child, and impacting the child’s emotional regulation and own vulnerability to mental health problems [20]. However, Stein et al. [2] suggest that such risks are not inevitable, with parenting emerging as a “key modifiable pathway”.

Improving parental mental health offers a valuable potential pathway for intervention to address and improve parent-child relationships and interactions, thus also improving child outcomes in the long term [21,22]. Specific factors related to parent-child interactions include parental attention and contingent responsiveness to infant cues, the ability to think about and appreciate the child’s perspective, thoughts, and feelings, as well as provide support to the infant when distressed [23,24]. Adults who are able to form and maintain good relationships can also parent effectively and are less likely to be dependent on health supports (particularly mental health) both for themselves and for their children [25]. However, the ability to mediate risks to the parent-child relationship becomes more difficult in situations where there is poverty, poor practical and financial support, and poor social support, including support from a partner [26]. It is therefore necessary to consider such external factors when designing and evaluating interventions. Additionally, as the current study setting has a high prevalence of such risk factors, a trauma-informed parenting intervention that can target more modifiable behaviours is critical.

### Parenting interventions and outcomes for children

Evidence-based early parenting interventions are important for minimising the risks associated with poor outcomes in children [27]. Effective parenting interventions often include elements to encourage responsive caregiving [28] as well as elements from the Nurturing Care Framework [27,29].

Mellow Parenting (MP) is an attachment-informed, group-based intervention programme that was developed in Scotland to support successful relationships between caregivers and their children [30,31]. MP works with children and their families who are living under multiple adversities, such as poverty, drug dependency, and parental mental health problems [32]. This approach provides a range of attachment-based parenting programmes that are “culturally adaptable, flexible to local needs, and have produced good results” and has been adopted in a number of countries around the world [33]. MP supports parents in improving responsive interactions with their child while also helping them to improve their own wellbeing and parenting confidence [34]. This aspect of the intervention is key as poor mental health in parents can lead to impaired cognitive development of infants, behavioural problems and increased risk of mental health problems later in life [35,36]. The impact of the MP programme has been shown not only to be advantageous for the child, but also helpful for the improvement of parental mental health [30]. The current study focuses on two current MP programmes - Mellow Babies (MB) and Mellow Toddlers (MT) - which have been delivered in Moldova since 2016. MB provides support for carers of infants and babies 0–18 months old, and MT supports families with children in the range from 19 months–5 years.

### Mellow parenting in Moldova

Moldova is an Eastern European state with an upper middle-income status. It is largely dependent on agriculture, with almost 60% of its population living in rural areas. Although Moldova’s economy has grown over the last twenty years, unemployment rates are typically high and poverty remains pervasive, particularly in rural areas [37]. A decreasing, and ageing, population has led to reduced production and reliance on social welfare for low-income households [38]. Moldova has experienced increasing external migration since 1991 as a result of the collapse of the Soviet Union with residents moving abroad for work due to economic instability [39]. This has led to many “left-behind” children being cared for by one parent, grandparents, or extended family members, with 1 in 4 children having at least one parent living abroad [40]. There are multiple barriers in Moldova for at-risk children, especially around accessing a secure and nurturing home. Children living in poverty or whose caregivers are economic migrants were identified by situational analysis as at-risk groups for neglect [41].

Historically, there has been a lack of government policies in Moldova designed to support parents in caring for their children and keeping families together. State provision was focused on institutional care for children, and parents would regard institutionalisation as a better option than maintaining children at home under difficult circumstances [40].

While deinstitutionalisation has been an important focus of care reform efforts to date, there is a critical need to secure robust family support services to ensure children are cared for in safe and nurturing families. Despite the significant progress in advancing childcare reforms in Moldova, more effort is needed to strengthen organisational capacity at local level to provide support to families and ensure optimal outcomes for children [41]. Strengthening parental skills and improving the wellbeing of children continue to be one of major priorities of the present National Child Protection Programme of the Republic of Moldova (2022–2026).

Partnerships for Every Child (P4EC; https://www.p4ec.md), a Moldovan Non-Governmental Organisation, in operation for 25 years, has supported the Government of Moldova to reform the childcare system, including legislation change, development of preventive and specialised services/programmes, and capacity-building of child welfare practitioners nationwide. It has implemented a range of developments aiming to reform how children are cared for, grounded within an ethos of implementation of UN Guidelines for the alternative care of children. One area of activity is focused on supporting moves away from institutional-based childcare and returning children to their families and the community [40], as well as the development of family strengthening services, such as parenting intervention programmes. In recognition of the lack of early intervention for at-risk children, P4EC partnered directly with Mellow Parenting to identify implementation partners in Moldova. Mellow Parenting was introduced in Moldova in 2016 with Mellow Parenting UK implementing the intervention in collaboration with Moldovan local authorities (see Methods below).

### Evidence base for Mellow Parenting

There is a small but growing body of research into the effectiveness of MP. A meta-analysis of eight studies [42] reported that MP programmes were associated with improved maternal wellbeing and reduced child behaviour problems, both of medium effect size, although there were challenges related to the small number of studies analysed, heterogeneity of data and limitations on the analysis because of unavailable data [42,43]. Subsequent studies have found that following participation in Mellow Parenting programmes, there were statistically significant improvements in maternal mental health and wellbeing, in parenting competence, and in the reported conduct problems of children [30]. Similarly, Raouna et al. [32] reported that parental anxiety and psychological distress were reduced after taking part in the Mellow Babies programme. Parents’ perception of their closeness of their relationship to their child was shown to increase alongside confidence in their parenting abilities. Families who had higher risk profiles benefited significantly from completing the programme, and up to 58% of families who were involved with child protection services experienced a de-escalation of their case. However, both of these studies were uncontrolled. A small RCT study [44] suggested that although no improvement was observed in parental mental wellbeing scores, there was a significant increase in positive parent-infant interactions after taking part in the Mellow Babies programme. Null findings may be due to levels of mistrust pre-intervention, with mothers underreporting their mental health symptoms initially, while showing more accurate self-reporting as the intervention progressed [44], which in itself may denote an improvement in parental mental health awareness. This aligns with studies suggesting that many mothers feel uncomfortable initially in reporting anxiety or depressive symptoms to health professionals due to fear of stigmatisation and perceived judgement [45]. All three studies were carried out across the United Kingdom, although many of the parents who took part in the programmes were considered to be ‘at-risk’ due to their lived experiences with social adversity [32,44].

Internationally, MP has been implemented in New Zealand and, in particular focusing on socially disadvantaged indigenous Māori families, has been well received. Qualitative data from New Zealand reported an increase in maternal wellbeing and parenting confidence, as well as a reduction in perceived children’s problematic behaviours and increased social skills [46]. Implementation of both online and in-person versions of MP in countries within eastern Europe and Central Asia, have also returned significant preliminary results. Feasibility studies suggest that within Türkiye and Tajikistan, MP programmes significantly increase maternal wellbeing, reduce parental stress, and improve the mother-infant relationship [47–49]. These results suggest it is feasible to deliver MP across multiple countries, in groups with identified needs for parenting support, albeit with different cultural parenting practices.

### Aims, research questions and hypothesis

The current study investigated the association between implementation of MP interventions in Moldova and both parental mental health and parenting outcomes, specifically: wellbeing, parenting confidence, and parenting daily stress.

The primary research question was: Do parents who attend Mellow Parenting experience improvements in their wellbeing?

Additionally, do parents who attend Mellow Parenting demonstrate improvements in parenting confidence; and do parents who attend Mellow Parenting experience improvements in parenting daily stress?

Based on the existing literature [32,47,48] it was hypothesised that parents who completed Mellow Parenting would demonstrate improvements in parenting wellbeing and parenting confidence and a reduction in parenting daily stress.

## Methods

### Design

The study was a pre-post non-controlled evaluation of parents’ wellbeing, confidence, daily stress and impact on the child following Mellow Parenting group intervention. It comprised a secondary data analysis of a dataset of outcomes measures from intervention groups run in Moldova between 2016 and 2020. Due to the collection of real-world data as part of the evaluation process of intervention groups, no control group was included in the data collection. Data for analysis were accessed for research purposes on 1 October 2022. The study authors had no access to any information that could identify individual participants within the study. Ethical approval for the current study was provided by the University of Edinburgh Ethics Committee.

### Participants

Parents participating in the Mellow Parenting groups were eligible if they:

1. Had a child aged between 0 and 5 years old (Mellow Babies age range is 0–18 months, Mellow Toddlers age range is 19 months–5 years).
2. Were in contact with that child during their participation in the group.
3. Could bring their child to the MP group for a lunchtime activity.
4. Be willing to participate in, and consent to, video feedback.

Mellow Parenting practitioners facilitated MP groups of parents recruited from nursery schools, kindergartens and family centres, as well as families who were registered as “at-risk” within the Social Work Department. Families deemed “at-risk” included those experiencing poverty, presenting with mental health difficulties, lacking access to services, deinstitutionalised children, or exposure to neglect or domestic violence. Overall, the families faced a wide range of complex and interrelated challenges. A total of 251 parents were involved in this study: n = 244 mothers and n = 7 fathers. Participants were allocated to Mellow Babies or Mellow Toddlers programmes depending on their child’s age. The majority of the Mellow Parenting groups were recruited and delivered in rural towns. However, groups were also recruited and facilitated in the capital city, Chișinău.

Attendance rates were high across all groups. Mellow participants who attend at least 70% of the group sessions (i.e., at least 10/14 sessions) are considered program completers [32]. A total of 226 participants attended at least 70% of sessions, 131 of whom attended 100% of the sessions. Seven participants attended fewer than 70% of the sessions. Additionally, 18 participants did not have their attendance recorded.

### Measures

#### Adult Wellbeing Scale (AWS).

The Adult Wellbeing Scale (AWS; [50]) is an 18-item self-assessment questionnaire. It was created to provide an appropriate measure for adult wellbeing that included the assessment of irritability in adults. Four subscales are explored through the Adult Wellbeing Scale; (1) depression, (2) anxiety, (3) inwardly directed irritability and, (4) outwardly directed irritability. Respondents are asked to rate their response to questions on a 4-point Likert scale. The scale has been recommended for use in research and clinical settings. Cut off scores may indicate problematic or increased symptoms for each scale (see Table 1 for participants scoring above the cutoff). The AWS is reported to be a well validated scale with alphas ranging from α = .71−.81 in previous studies of women taking part in early parenting programmes [51].

**Table 1:** Demographic data for all participating mothers.

Study variable	Frequency (%)	Mean (SD)
**Age**	**(N** ** = 120)**	29.43 (7.08)
16–24	34 (28.3)	21.18 (2.41)
25–29	31 (25.8)	27.53 (1.43)
30–39	41 (34.2)	33.68 (2.99)
40–48	14 (11.7)	42.23 (2.39)
**Other children**	**(N** ** = 84)**	
No other children	35 (41.7)	
Other children	49 (58.3)	
**Nationality**	**(N** ** = 112)**	
Moldovan	108 (96.4)	
Ukrainian	3 (2.7)	
Russian	1 (0.9)	
**Relationship status**	**(N** ** = 118)**	
Married/cohabiting	78 (66.1)	
Single	33 (28)	
Divorced/Separated/Widowed	7 (5.9)	
**Employment**	**(N** ** = 115)**	
Employed – full-time	11 (9.6)	
Employed – part-time	5 (4.3)	
Unemployed – on benefits	2 (1.7)	
Unemployed – not on benefits	18 (15.7)	
Unemployed – benefit status unknown	79 (68.7)	
**Education**	**(N** ** = 118)**	
Did not finish school	12 (10.3)	
Still in school	4 (3.4)	
Finished school	75 (64.1)	
Higher education	26 (22.2)	
**Adult Wellbeing Scale (AWS)**	**(N** ** = 236)**	
Increased depressive symptoms	91 (38.5)	
Increased anxiety symptoms	64 (27.1)	
Increased outward directed irritability	32 (13.6)	
Increased inward directed irritability	24 (10.2)	
**Strengths and Difficulties Questionnaire (SDQ)**	**(N** ** = 145)**	
Total difficulties (High/Very High)	84 (57.9)	
Emotional difficulties (High/Very High)	77 (53.1)	
Conduct difficulties (High/Very High)	63 (43.4)	
Hyperactivity (High/Very High)	33 (22.8)	
Peer difficulties (High/Very High)	86 (59.3)	
Prosocial behaviours (Low/Very Low)	52 (35.9)	
**Parenting Daily Hassles Scale (PDHS)**	**(N** ** = 160)**	
Minor stressor events experienced	42 (26.3)	
Frequent feelings of stress experienced	29 (18.1)	
**Karitane Parenting Confidence Scale (KPCS)**	**(N** ** = 77)**	
Non-clinical parenting confidence	22 (28.6)	
Mild low parenting confidence	18 (23.4)	
Moderate low parenting confidence	13 (16.8)	
Severe clinical low parenting confidence	24 (31.2)	

#### Strengths and Difficulties Questionnaire (SDQ).

The Strengths and Difficulties Questionnaire for 2- to 4-year-olds (SDQ; [52]) measures a child’s behaviours, social interaction, and emotional functioning. There are five items that the SDQ looks to measure: conduct problems, emotional symptoms, hyperactivity, peer relationships, and prosocial behaviour. Respondents are asked to rate their response to 25 questions which relate to the subscales on a 3-point Likert scale: (1) Not True, (2) Somewhat True, (3) Certainly True. Completed scores rate children as Close to Average, Slightly Raised/ Slightly Lowered, High/ Low, Very High/ Very Low depending on the subscale (see Table 1 for High/ Very High cutoffs). The SDQ has been shown to have a good test-retest reliability, good external validity and moderate-to-strong internal reliability across the five measures, cross culturally and across translations [53]. Within this study, the SDQ was utilised with participants attending Mellow Toddlers (i.e., 12–60 months), however, as some of the toddlers’ ages were not reported, it is unclear whether all the children met the two-year-threshold, which could limit the validity of the scores, and as such, the results from the SDQ should be interpreted with caution.

#### Crnic Parenting Daily Hassles Scale (PDHS).

The Crnic Parenting Daily Hassles Scale (PDHS; [54]) is a 20-item self-report measure that investigates how frequently and to what intensity parents experience routine stressor events that are associated with caregiving. The scale was created due to the lack of assessments available for daily parenting ‘hassles’, as all the available measures assessed more substantial stressor events [54]. Frequency is measured on a 4-point Likert scale. Parents are then asked to rate the intensity of the hassle on a scale of 1–5. Scores relating to frequency are totalled and scores over 50 are indicative of high likelihood of minor stressor events taking place. If the sum of the intensity scores is 70 or above, this indicates that the caregiver is experiencing frequent feelings of stress in their caregiving (see Table 1). The PDHS has been reported as reliable across diverse parenting populations [55,56]. As the PDHS is validated for use in toddlers, data were only available for participants whose child was aged >18 months old.

#### Karitane Parenting Confidence Scale (KPCS).

The Karitane Parenting Confidence Scale (KPCS; [57]) is a 15-item self-report questionnaire. The purpose of the measure is to rate perceived parental self-efficacy and is targeted at parents of children who are between 0 and 12 months. The KPCS comprises three subscales: (1) Perception of parenting ability, (2) Available parenting support, (3) Perceptions about child development. Respondents are asked to rate their answers to the question on a 4-point Likert scale. The KPCS score places the caregiver in one of four categories in terms of low parenting confidence: non-clinical (> 40), mild (36–39), moderate (31–35) or severe clinical (< 31) range (see Table 1 for participant cut offs). The KPCS has been proven to be robust in internal validity and to have test-retest reliability [57,58] and is recommended for use in both clinical and research settings. These data were only available for participants whose child was aged <18 months old.

### Intervention

The MP interventions were divided into two categories: Mellow Babies and Mellow Toddlers. Both groups follow the same format. The intervention was delivered over 14 weeks with one session taking place each week. Each weekly group lasted 4.5 hours and was split into three sessions: morning, lunchtime, and afternoon. To ensure the programme was accessible, programme materials were translated into Romanian. Minor editorial adaptations to materials were made to reflect cultural and linguistic contexts (e.g., names, titles). As the content was designed to initiate discussion and reflections based on the lived experience of group participants, no further changes were made to the materials as this did not require cultural adaptation. In the morning session, parents reflected on their own current situation and also on childhood experiences. They explored what impact this might have on their current relationship with their child. The afternoon session focussed on supporting parents to develop a strong relationship with their baby or toddler. The group content in the afternoon workshops was modified to be appropriate for the child’s stage of development. These sessions used video recordings of interactions between the parents and their child to provide specific and constructive feedback to the parents. The intervention was carried out in the same facility from which parents were recruited. A children’s “nurture group” was run at the same time as the adult sessions so that childcare was not a barrier for attendance. Depending on the children’s ages, the nurture group offers them opportunities to learn, socialise with peers, and rest. A key component of the programme is the time spent together for lunch and a shared activity. This is when parents and children come together to enjoy lunch and participate in shared activities such as singing, reading, painting and playing games.

The development of the MP programme supported the implementation of one of the Government priorities on strengthening of carers’ capacity to raise and take care of their children by developing programmes for strengthening parental skills, which was aligned to the existing inter-agency strategies on child protection (2014–2020) and enhancing parenting skills (2016–2022). Mellow Parenting groups were co-ordinated by P4EC a Moldovan non-governmental organisation. Mellow Parenting UK was sponsored to train local practitioners with the support of translators and to develop some practitioners to supervisor level. In-person training on Mellow Programmes was facilitated by staff from MP and the criteria for becoming a Mellow practitioner in Moldova included staff from local authorities with experience of working in early years settings and experience of working with parents. As part of the Mellow training, practitioners were encouraged to share their personal experiences with participants and to encourage the parents to devise their own solutions to problems. Between 2016–2021, staff from Mellow Parenting (UK) provided five training events, each including a 4-day in-person training course, which included practical advice on how to set up the programme, training on working with participants to build relationships within the group setting and encourage responsive parenting, as well as an introduction to attachment theory (critical to the programme’s theory of change).

Over 30 practitioners have been trained in Mellow programme delivery in Moldova during 2016–2021. During and after completion of delivery of the programme, Mellow practitioners in Moldova were able to advance their training through supervision and Reflective Consultations with Mellow Parenting trainers, allowing them to achieve accreditation status. A small number of practitioners also trained to become trainers themselves, building local capacity for sustainability of the parenting programme. P4EC in partnership with Mellow Parenting through the funding from the European Union and World Childhood Foundation, provided structured support to the national and local authorities (LAs) to set up the Mellow Parenting programme and engaged with various stakeholders to build capacities, strengthen coordination, develop and strengthen policies and services regarding the early years parenting programmes.

Practitioner training was provided for the delivery of the group materials. This included workshops on how to administer the evaluation materials and submit results to MP. Practitioners were allocated to supervisors for support and to ensure that groups were delivered with fidelity. Supervisors were also given assistance in the collection of data from the groups. Data collection was handled by practitioners within the local authorities, and representatives from P4EC. Pre-intervention measures were administered to parents before session one by the group practitioners, who also collected post-intervention measures, either after sessions 13 or 14. At times, post-intervention measures were collected after the last attended group meeting during a home visit.

### Analysis plan

All data analysis was carried out using SPSS v28. Intervention effects were assessed through analysis of the pre- and post-measures. Changes from before to after intervention were explored by using the main effect of time, on the outcome measure of total scores of the pre- and post-MP intervention for all four outcome measures (AWS, SDQ, PDHS and KPCS).

All participants who were mothers were included in the data analysis (n = 244). As there were only seven fathers in the dataset, these were excluded from the subsequent analyses in order to maintain relative homogeneity of the sample. As such, the current study focused on the impacts of Mellow Parenting on mothers only, and the results may not be generalisable to fathers’ parenting practices in Moldova. Data from Mellow Toddler and Mellow Baby groups were initially combined to assess the outcome of the Mellow Parenting Intervention. Differences between groups were explored using Analysis of Covariance (ANCOVA). The impact of the MP intervention on the outcomes (change of score between pre- and post- measures) was analysed using the main effect of time.

For participants with complete data, two-tailed paired-samples t-test were administered for each of the outcome measures to compare changes in parent’s pre- and post-intervention. For non-normal data Wilcoxon signed-rank tests were used as a sensitivity analysis. There were no changes in significance using the Wilcoxon signed-rank test, therefore, t-test results are reported in the results.

Correlational analyses were performed on participants’ age and programme attendance rates, against each of the pre-scores of the outcome measures to assess for any significant differences in participants before they began the MP intervention. ANCOVAs were run on the nominal datasets to assess whether these demographic differences had a significant effect on outcome measure pre-scores prior to intervention taking place. Whether a parent had a child living in the house or in care was disregarded as the number of children living outside of the family home was minimal (n = 3).

A sensitivity analysis was conducted to explore whether there were outcome differences between the Mellow Babies and Mellow Toddler programmes. Analysis of covariance (ANCOVA) was carried out on adult wellbeing scores and parenting confidence as these were the two scales that participants in both groups had completed. However, group numbers were too low for Mellow Babies (n = 9) to assess if there was a significant difference between the groups. Therefore, a sensitivity analysis could only be run on parenting wellbeing.

## Results

### Demographic data

Within the sample, 54.2% (n = 65) of mothers participating in the MP intervention were under the age of 30 years old. The youngest mother to participate in the group was 16 years and the oldest was 48 years. Only 13.9% (n = 16) of mothers in the group were in any form of employment. Of the remaining 86.1% (n = 99), who were unemployed, there was insufficient data to identify how many were in receipt of benefits. For full demographic data, and cut off scores for measured variables, see Table 1.

### Impact of group intervention (pre- post-intervention scores)

Significant pre-post intervention improvements were found on AWS total scores (t(239) = 11.49, *p* < .001) with maternal total wellbeing statistically improving over the intervention. Significant changes were also observed across AWS subscales with scores for depression (t(239) = 5.95, *p* < .001), anxiety (t(239) = 5.95, *p* < .001), outward directed irritability (t(239) = 6.96, *p* < .001), and inward directed irritability (t(239) = 8.50, *p* < .001) all statistically decreasing after intervention.

Total scores on the SDQ significantly reduced after intervention (t(146) = 4.06, *p* < .001) indicating that the overall child behaviours that parents perceived as having a negative impact had reduced. A reduction in parents’ perceptions of their child’s emotional symptoms was significant after intervention (t(146) = 2.41, *p* = .017). Scores for conduct problems (t(146) = 3.76, *p* < .001), and hyperactivity (t(146) = 5.34, *p* < .001) significantly improved after attending the MP programme. This was not the case for perceptions of peer problems, which did not significantly reduce post intervention (t(146) = 1.06, *p* = .289).

Parents’ perceptions of hassle frequency (t(155) = 4.20, *p* < .001) and hassle intensity (t(157) = 6.13, *p* < .001) both significantly improved after parents attended MP. Parents showed a significant improvement in their perception of their child’s challenging behaviour (t(157) = 5.64, *p* < .001) as well as their ability to carry out parenting tasks (t(157) = 5.92, *p* < .001). Parenting confidence (t(76) = −7.85, *p* < .001) significantly increased post intervention.

Alpha correction was used to adjust for multiple comparisons with *p* = .003. All results remained significant apart from parents’ perceptions of child’s emotional symptoms, which was no longer significant (Table 2).

**Table 2:** Change in outcome measures before and after group intervention.

Outcome measure	Sample N	Mean baseline scores (SD)	Mean change in outcome scores (SD)	Standard error mean	95% CI (lower, upper)	Sig. (2-tailed)
AWS^a^ Total	236	21.34	4.90 (6.61)	0.43	4.06, 5.75	**<.001**
AWS Depression	236	5.87	0.87 (2.26)	0.15	0.58, 1.15	**<.001**
AWS Anxiety	236	7.09	1.79 (2.65)	0.17	1.46, 2.13	**<.001**
AWS Outward Irritability	236	5.02	1.05 (2.33)	0.15	0.75, 1.34	**<.001**
AWS Inward Irritability	236	3.47	1.20 (2.20)	0.14	0.93, 1.48	**<.001**
SDQ^b^ Total Difficulties	145	16.73	2.24 (6.68)	0.55	1.15, 3.33	**<.001**
SDQ Emotional Symptoms	145	3.59	0.49 (2.46)	0.20	0.09, 0.89	.017
SDQ Conduct Problems	145	3.93	0.74 (2.37)	0.20	0.35, 1.12	**<.001**
SDQ Hyperactivity	145	5.38	0.86 (1.96)	0.16	0.54, 1.18	**<.001**
SDQ Peer Relation Problems	145	3.88	0.20 (2.25)	0.19	−0.17, 0.56	0.289
SDQ Prosocial Behaviour	145	6.16	−1.22 (2.14)	0.18	−1.57, −0.88	**<.001**
PDHS^c^ Hassle Frequency	160	43.11	2.55 (7.59)	0.61	1.35, 3.75	**<.001**
PDHS Hassle Intensity	160	54.59	7.27 (14.9)	1.19	4.93, 9.62	**<.001**
PDHS Challenging Behaviour	160	20.73	2.70 (6.03)	0.48	1.76, 3.65	**<.001**
PDHS Parenting Tasks	160	22.76	3.13 (6.63)	0.53	2.08, 4.17	**<.001**
KPCS^d^ Total	77	34.34	−5.09 (5.69)	0.65	−6.38, −3.80	**<.001**

*p* values reported in **bold** are statistically significant after Alpha correction.

^a^WS: Adult Wellbeing Scale;

^b^SDQ: Strengths and Difficulties Questionnaire;

^c^PDHS: Parenting Daily Hassles Scale;

^d^KPCS: Karitane Parenting Confidence Scale.

### Impact of attendance and participants’ age on outcomes

The relationship between participants’ age and attendance rates and the outcome measures pre-scores was explored using correlational analyses (Table 3). Emotional symptoms and peer problems were excluded from the analysis as they did not reach statistical significance in the t-tests. No significant relationship was found between participant age and pre-intervention outcome measures. Correlational analysis suggested that participants who rated themselves as having higher inward irritability, or who perceived their children to have higher rates of conduct problems, had higher attendance (*p* < .05*).* However, after applying a Bonferroni adjustment, (where *p* = .003), no significant relationship was found between participant attendance rates and pre-intervention outcome measures.

**Table 3:** Correlation table for participants’ age and attendance rates with outcome measures pre-scores.

	1	2	3	4	5	6	7	8	9	10	11	12	13	14	15
1 Age															
2 Attendance	−.053														
3 AWS total	.049	.115													
4 AWS depression	.029	−.056	.604**												
5 AWS anxiety	.000	.105	.760**	.402**											
6 AWS outward irritability	−.040	.124	.741**	.217**	.398**										
7 AWS inward irritability	.070	.135*	.591**	.142*	.314**	.376**									
8 SDQ total	−.076	.141	.197*	−.019	.195*	.251**	.192*								
9 SDQ conduct problems	−.005	.180*	.194*	−.004	.187*	.244**	.190*	.770**							
10 SDQ hyperactivity	−.101	.089	.179*	.026	.181*	.214**	.128	.708**	.443**						
11 SDQ prosocial	.000	−.093	.036	−.038	−.012	.005	.038	−.052	−.058	−.087					
12 PDHS frequency	.172	−.018	.366**	.141	.300**	.395**	.135	.321**	.324**	.224*	−.062				
13 PDHS intensity	.068	.052	.418**	.179*	.432**	.376**	.149	.125	.124	.055	.027	.529**			
14 PDHS challenging behaviour	.105	.023	.398**	.150	.425**	.365**	.134	.181*	.156	.087	−.011	.542**	.912**		
15 PDHS parenting tasks	.050	.093	.412**	.211**	.400**	.386**	.157*	.149	.150	.090	−.034	.493**	.930**	.837**	
16 KPCS	−.059	−.247	−.427**	−.143	−.321**	−.424**	−.239*	^c^	^c^	^c^	^c^	^c^	^c^	^c^	^c^

*p < .05; **p < .01.

^c^Cannot be computed because at least one of the variables is constant.

### Impact of other demographic factors

**Fig 1:**
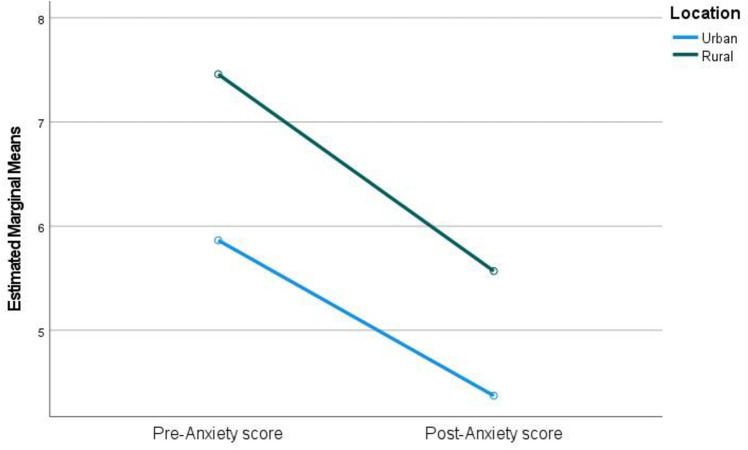
Pre- and post-anxiety scores for urban and rural groups.

There was a statistically significant interaction between urban and rural groups and post-intervention anxiety ratings after controlling for pre-intervention anxiety outcomes (F(= 4.61, *p* = .033, partial ƞ² = .019). Anxiety scores for rural participants were higher than that of urban groups when pre-scores were collected. Although scores were reduced for both groups, a greater reduction was seen in the rural group after intervention (Fig 1).

**Fig 2:**
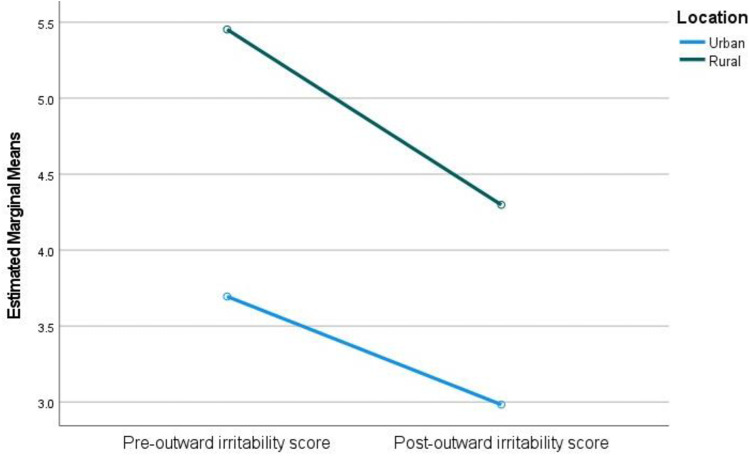
Pre- and post-outward irritability scores for urban and rural groups.

A statistically significant interaction was also found between urban and rural groups and post-intervention outward irritability ratings while controlling for pre-outward irritability outcomes (F(= 3.99, *p* = .047, partial ƞ² = .017). Pre-intervention outward irritability scores were higher for the rural group than the urban group. Scores reduced for both groups; however, a greater reduction was seen in the rural group (Fig 2).

**Fig 3:**
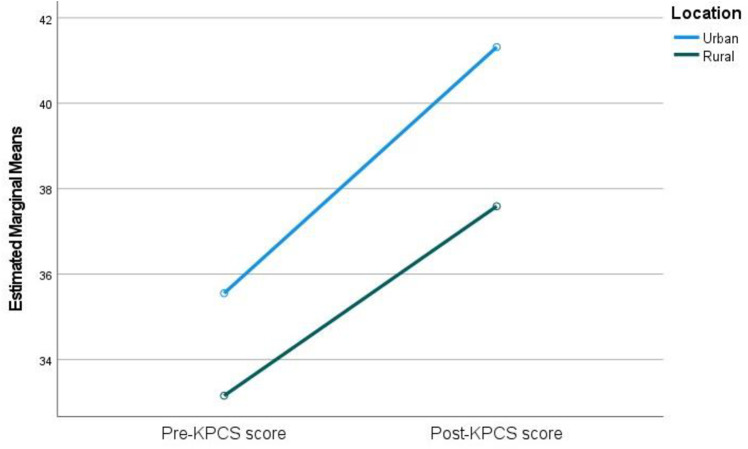
Pre- and post-confidence scores for urban and rural groups.

Post-intervention parenting confidence scores had a statistically significant interaction between urban and rural groups, while controlling for pre-intervention parenting confidence scores (F(= 8.49, *p* = .005, partial ƞ² = .103). Parenting confidence scores were higher pre-intervention for the urban group than for the rural group. The improvement of parenting confidence in the urban group was greater than for that of the rural group (Fig 3).

No statistically significant interactions were found between outcome measures and nationality, education, relationship status, employment status, and differences in the number of children participants had. An analysis was not run on whether there was a difference between parents whose child was being cared for in the home or out of the home due to uneven group sizes (children cared for outside of home n = 3). A sensitivity analysis was conducted to explore whether there were outcome differences between the Mellow Babies and Mellow Toddler programmes. No significant difference was found between those who participated in the Toddlers or Babies programme.

## Discussion

### Summary of findings

This study identified that Moldovan mothers who completed a Mellow Parenting intervention programme reported significant improvements in their wellbeing and confidence as well as reduced parenting daily stress. This is consistent with previous studies’ reporting that participation in MP programmes improves the mental wellbeing of parents as well as their ability to cope with daily parenting tasks [30,32,46]. Parenting stress was seen to reduce for participants after completion of the intervention. There was also a marked improvement in parenting confidence with average participants’ scores reducing from the ‘moderately clinical’ range to the ‘mildly clinical’ range. This is consistent with evidence that parenting programmes which increase parenting self-efficacy can lead to a reduction in stress [59].

In terms of the impact of the parenting programme on children, there was a reduction in the overall SDQ scores indicative of a perceived improvement in the child’s behavioural outcomes. The average score for participants dropped from the ‘high’ range in pre-intervention scoring to ‘slightly raised’ range post intervention. There was a significant improvement in perceived conduct problems and hyperactivity subscales. However, there were no significant differences in the mothers’ perception of the child’s emotional state or the perception of peer problems. These findings are also consistent with Bloomfield and Kendall’s [59] assertion that coping with a child’s behaviour is one of the main motivators for a parent attending a parenting group and so this is potentially where the impact is more likely to be noticed. These findings also support previous research indicating that improvements in parental mental health outcomes are associated with improved child behavioural outcomes, particularly externalising behaviours [17].

Mellow Parenting is an attachment-based programme, but positive results were mainly related to behavioural issues, with the intervention appearing to be more instrumental in addressing parenting behaviours and parents’ perceptions of outwardly directed difficulties. There were no significant changes to emotional symptoms as parents were not reporting they felt more attuned to their child’s feelings or that their child’s social relationships had improved. This may be because these traits are more difficult for parents to observe or that the tools used to measures such constructs are less sensitive to these characteristics. It is also important to take into account the impact of environmental stressors and the quality of relationship between a child’s caregivers on the quality of attunement and parent-child interactions [60]. Potential difficulties that mothers were facing at home such as domestic violence, which has a high prevalence in Moldova, could have impacted on the quality of attunement and attachment, impeding the efficacy of the intervention [61].

Demographic data showed that mothers who participated in the group represented heterogeneity across certain features, such as education, and geographical locations. However, the majority of participants were married or cohabiting with a partner, and additionally, the majority were unemployed even though many were educated to the point of finishing school or even higher education. Given that participants were mothers of young children, this could indicate a traditional family and social structure where women retain their roles as homemakers and do not enter into employment. However, the current study had insufficient information regarding reasons for participants’ unemployment.

Overall, MP appears to be a robust intervention within Moldova, although there was some variation on the effectiveness of the programme between certain demographic groups. Comparing the results of urban and rural groups, parenting outcomes for both populations improved but there were significant differences in the effect sizes across groups. In terms of parental anxiety, rural participants started with higher self-reported levels of anxiety than participants in the urban groups. The rural group, however, saw a more dramatic improvement in their anxiety scores in comparison to the urban group. Similarly, outward irritability was initially higher for rural groups and there was a greater improvement in their levels of irritability in comparison to the urban participants. Higher levels of anxiety, stress, and lower parenting confidence in rural populations could be a result of rural poverty and isolation and the majority of mental health resources and social supports being concentrated in urban areas [62]. Urban groups, conversely, may have had better access to facilities and a better mental health literacy. In terms of parenting confidence, urban participants started with higher levels of confidence and also had a larger improvement in confidence in comparison to the rural groups. McManus & Poehlmann [26] suggest that environmental factors such as poverty and poor financial and social support can cause difficulties when mediating risks associated with parent-child outcomes. This is reflected in the pre-intervention scores. However, although rural groups reported worse outcomes pre-intervention, the impact of the intervention appeared to be greater for them in terms of anxiety and outward irritability. Longitudinal follow-up data would be desirable to assess whether intervention improvements are sustained.

### Limitations

As the data for this sample were collected in a ‘real world’ setting, there was no control group included in the data collection, so a causal relationship between the changes observed and MP groups cannot be definitively inferred. Inclusion of a control group in future studies would be important. It was also unclear to what extent translated measures were culturally appropriate for the study. As the measures used were largely created and tested on UK and US populations there is limited research on the validity for mothers and children in Moldova and whether the measures take into account cultural sensitivities. Another limitation was the lack of child outcome data. The SDQ was used as a measure for the toddler groups, however, it still requires adequate validation within this age group [63]. Observational measures could be considered which may provide more context about the lack of change in the attunement-based measures.

### Implications for practice and further research

This is the first study to assess the effectiveness of the MP programme in Moldova. The results of this study are important in understanding the impact of MP on this population and the cross-cultural applicability of the programme. MP was developed in Scotland, with a less traditional parenting culture than in Moldova and without having the same parental migration issues [64]. The result of the study suggests that regardless of fundamental cultural differences, MP still produced positive outcomes for both parents and children in Moldova. This is in line with several other MP studies in Tajikistan [47,49] and New Zealand [46] which reported positive outcomes for both mothers and children in at risk communities.

The results from this study are especially relevant to the concerns that UNICEF [65] and P4EC [40] recognised in terms of increasing early intervention support for at-risk families in Moldova to improve child outcomes, as well as the commitments to ‘at-risk’ families reflected in the Moldovan Government’s National Child Protection Programme (2022–2026). Reaching vulnerable populations remains a complex challenge for social work professionals, especially when engaging families in programmes like Mellow Parenting that go beyond traditional financial or material aid. In many communities, especially disadvantaged or rural ones, direct aid is still the most familiar and expected form of support. As a result, initiatives such as parenting groups or psychoeducational programmes are often met with hesitation or scepticism. Group-based interventions, such as Mellow Parenting, are relatively new models of support in these types of settings. Families may fear being judged or blamed for their parenting, or may perceive participation as a waste of time—particularly if they do not immediately understand the benefits of participation. These perceptions are often compounded by deeper issues such as lack of trust in institutions/services, stigma around seeking help, and limited awareness of additional support services. To address these barriers effectively, a coordinated effort is needed among government actors, civil society organisations, and community-based services. Scaling and sustaining such models requires not only training professionals in programme delivery and core principles, but also promoting a cultural shift. This includes raising awareness about the value of parenting support, implementing outreach strategies sensitive to local contexts, and embedding group-based programmes within broader family support services.

Findings from the study also suggest that MP may be an appropriate intervention for other countries in Eastern Europe that face similar socio-geographical issues as Moldova. Further, since the 2022 Russian invasion of Ukraine, over 1,200,000 Ukrainian refugees have entered Moldova, either as a staging point or as a final destination [66]. The number remaining in Moldova is around 120,000 [66]. Many of these refugees will be women and children and Moldovan resources are limited. The support that the Moldovan government is giving to parents and young families will be further stretched to include refugees who bring with them particular financial, social, and emotional needs. When comparing urban and rural groups there was evidence that rural groups who had higher rates of anxiety and outward irritability saw a greater benefit from the intervention in their post-scores. Ukrainian refugees in Moldova are likely to have a higher baseline level of mental health difficulties and, while they will be experiencing very specific adversities, may show similar enhanced outcomes. Extending this study to the Ukrainian refugee population in Moldova may provide evidence as to whether MP is a useful approach for them [67].

Additionally, the results of this study suggest that MP is an effective early intervention programme. While MP is a relatively resource-intensive programme at implementation, the early intervention focus may provide long-term cost-effectiveness benefits as a support to families [68]. Recent feasibility studies have also suggested that hybrid or online versions of the intervention may be effective [48], and more research should be undertaken to understand if these formats provide the same results as in-person intervention albeit in a more cost-efficient setting.
